# Fabrication and Investigation of Deformable Rubber–Carbon Nanotube–Glue Gel-Based Impedimetric and Capacitive Tactile Sensors for Pressure and Displacement Measurements

**DOI:** 10.3390/gels10010076

**Published:** 2024-01-20

**Authors:** Khasan S. Karimov, Muhammad Tariq Saeed Chani, Tahseen Kamal, Syed Zameer Abbas, Naved Azum, Abdullah Mohamed Asiri

**Affiliations:** 1 Ghulam Ishaq Khan Institute of Engineering Sciences and Technology, Swabi 23640, Pakistan; khasan@giki.edu.pk (K.S.K.); zameer@giki.edu.pk (S.Z.A.); 2 Center for Innovative Development of Science and Technologies of Academy of Sciences, Rudaki Ave., 33, Dushanbe 734025, Tajikistan; 3 Center of Excellence for Advanced Materials Research, King Abdulaziz University, Jeddah 21589, Saudi Arabia; tkkhan@kau.edu.sa (T.K.); navedazum@gmail.com (N.A.); aasiri2@kau.edu.sa (A.M.A.); 4 Department of Chemistry, King Abdulaziz University, Jeddah 21589, Saudi Arabia

**Keywords:** carbon nanotube–glue composite gel, tactile pressure, capacitive sensing, impedimetric sensing, frequency

## Abstract

Carbon nanotube–glue composite gel-based surface-type elastic sensors with a cylindrical shape deformable (flexible) metallic body were fabricated for tactile pressure and compressive displacement sensing. The fabrication of the sensors was performed using the rubbing-in technique. The effect of the pressure and the compressive displacement on the capacitance and the impedance of the sensors were investigated at various frequencies (in the range of 1 kHz to 200 kHz). It was found that under the effect of pressure from 0 to 9 g/cm^2^, the capacitance increased by 1.86 and 1.78 times, while the impedance decreased by 1.84 and 1.71 times at the frequencies of 1 kHz to 200 kHz, respectively. The effect of displacement on the impedance and the capacitance of the device was also investigated at various frequencies from 1 kHz to 200 kHz. The results showed that under the effect of compressive displacement up to 25 µm, the impedance of the sensors decreased on average by 1.19 times, while the capacitance increased by 1.09 times, accordingly. The frequency response of the displacement sensor showed that it matched with the low-pass filter. The obtained results are explained based on changes in the shape and geometrical parameters of the cylindrical-shaped conductive body. These results have also been explained on the basis of the distance between the conductive plates of the capacitive sensors during compression, which takes place under the effect of applied pressure or displacement. Moreover, the design of the sensors is simple and easy to fabricate, and their use is also earthy. The fabricated sensors have great potential for commercialization.

## 1. Introduction

Over the past few years, the number of research articles published in the field of pressure sensing, especially related to the development of this area of electronic technology and devices, has increased [1,2,3,4,5,6]. For health monitoring and electronic skin applications, highly sensitive, flexible polymer transistors were fabricated and investigated by Schwartz et al. [7]. As an essential part of electronic skin, flexible pressure sensors permit robots and biomedical prostheses to naturally interact with the environment and humans. For these sensors, another striking application in longstanding medical diagnostics is mobile biomonitoring. Low-cost and durable micro force sensors (3D) were designed, fabricated, and tested for micro palpation (enhanced musical tuning) of biological entities [8]. These sensors have the capacity to interactively classify (via hearing) the biomarker or mechanical signature of the biological entities, including cells, tissues, embryos, and organs. These sensors have the ability to work as a “tactile stethoscope” that can measure the changes and properties of a biological entity by accessing its surface. A force and flexure-sensing device that can be embedded and compatible with a polymeric structure was fabricated for the polymeric mechatronics [9]. The development and evaluation of two significant moods of sensing for embedding in the mechanisms of robotic and polymeric mechatronics have been discussed. Here, IR phototransistors were used for the sensing of the joint angle multi-axis flexure, and silicon strain gages (embedded) were used for the sensing of a three-axis force and showed a performance like that of metallic element-based sensors of the same size (12 mm). For the robotic hand (MAPRoh-1), the movement of the finger was analyzed using a flexible bend and motion capture sensor [10]. This work was based on the analysis of the correlation between the produced angle (from the motion-captured system) and the voltage generated using the bend sensor that was attached to the robotic hand. Using flex sensors, flexing or bending can be measured with ease and low cost [11]. The robustness, compactness, low density, low power consumption, and effective measurement make these sensors attractive for various useful applications. To fabricate highly (100%) stretchable strain sensors, a cost-effective and easy technique was presented by Rahimi et al. [12]. This technique was based on the carbonized pattern’s embedment and transfer into the elastomeric substrates. The creation of these carbonized patterns was carried out by laser pyrolysis of thermoset polymers. These carbonized materials comprised CNTs (carbon nanotubes) and graphene particles (partially aligned) with a strident directional anisotropy that provided the base for the fabrication of highly stretchable and robust unidirectional strain sensors.

PDMS (polydimethylsiloxane) elastomer and carbon paper-based highly flexible strain sensors (resistive type) were fabricated by Li et al. [13]. The carbon paper used was prepared via the high-temperature pyrolysis of the tissue papers. These sensors showed ten times higher sensitivities compared to a traditional metallic strain gauge.

Similarly, because of their utilization in industrial monitoring and electronic devices (personal), pressure sensors have become a very strong contender for the promotion and progression of science and technology in modern society [14]. Organic materials-based pressure sensors, due to their low cost and high flexibility, are very attractive for wearable health monitoring devices and artificial intelligence systems. A flexible self-powered pressure sensor for noninvasive blood pressure and pulse wave measurement was fabricated by weaving and reported by Meng et al. [15]. These sensors showed ultra-high sensitivity (45.7 mV Pa^−1^) with a less than 5 ms response time (ultrafast) with the stability of the performance even after 40,000 motion cycles. For the precise monitoring of pulse waves from the ankles, wrist, ear, and fingertip, a low-power consuming sensor system was also developed. The measurements of these sensors were compared with commercially available cuff-based bp apparatus and observed with a 0.87 to 3.65 percent discrepancy. It was claimed that these sensors are cost-effective and efficient alternatives to currently available intricate cardiovascular monitoring systems. Ultrahigh-sensitive self-powered pressure sensors (triboelectric nanogenerators) for biological signal monitoring were fabricated using a mixture of polydimethylsiloxane and expandable microspheres through a simple and low-cost technique [16]. It was also reported that by adjusting the ratio of microspheres in the polydimethylsiloxane, various levels of sensitivity could be obtained. This sensitivity (at maximum) can be reached at 150 mV/Pa; hence, the monitoring of pulse and respiratory sensitivity can be performed by connecting these sensors with the wrist and chest of the human body accordingly.

As flexible sensors have widespread applications in humanoid robotics, including wearable electronics, smart prostheses, and health monitoring [17], the problems of flexible sensors are the stretching/bending strain and temperature drift, which are thought-provoking, and in practical applications, these issues may not be neglected. Wang et al. [17] suggested a monolithic integration of a compensating thermistor to overawe the negative effects of the bending/stretching strain and temperature in the flexible sensors. This compensating thermistor had the same material and a similar geometric shape as that of a thermistor (sensing) in a Wheatstone-bridge feedback circuit. The theoretical and practical (experimental) validation for the effectiveness of the suggested integration was also performed on the flexible flow and pressure sensors. As a result, the suggested compensation method was found to be effective in eliminating the strain and temperature effects and proved self-sustaining, fast, and easy to realize.

Liu et al. [18] fabricated graphite nanoplates and softwood fibers cellulose (Paper-based) flexible pressure and strain sensors using the paper-making technique and tested them without encapsulation under running water. For paper reinforcement and to make it super-hydrophobic, cellulose nanofibers were added, and the emulsion of the alkyl ketene dimer was coated on it, respectively. For pressure sensing, these sensors showed sensitivities of 0.019 kPa^−1^ and 0.01 for the pressure ranges of 0 to 316.5 kPa and 316.5 to 1421 kPa, respectively, with a response time of 0.3 s. While for strain sensing, the sensor’s gauge factor was up to 18.99, these sensors were considered for use in monitoring the elbow, wrist, and finger in different environments.

A device for the detection of target pressure was fabricated by Liao et al. [19], which consisted of a housing, membrane (resilient), and pressure detection unit. The housing forms a chamber with a through hole, while the resilient membrane is mounted to cover and open the housing to set the chamber in an airtight condition. A pressure sensor is in the pressure detection unit, which is mounted in the through hole and detects the movement of the floating element.

A review of the application of flex sensors was published by Alapati et al. [20]. Flexible ITO orange dye and CNT–Rubber-based sensors were fabricated using rubbing-in technology and investigated for pressure, temperature, displacement, infrared irradiation, and relative humidity sensing [21]. The sensing mechanism of these sensors was based on the change in impedance and resistance; under the effect of displacement up to 25 µm, the impedance and resistance were changed up to 1.34 and 1.62 times, respectively. While changing pressure up to 47 gf/cm^2^, the change in impedance and resistance were up to 1.34 and 1.35 times. The frequency response investigation showed that these sensors were high-pass filters with a 100 Hz cutoff frequency. A shockproof, flexible, superabsorbent polymer jelly and graphene-based electrochemical sensors were fabricated for temperature and humidity sensing [22]. On changing the temperature from 21 to 41 °C and the humidity from 47 to 98%, the change in the impedance was observed up to 2.4 and 2.0 times, respectively. The initial open-circuit voltages of these sensors were up to 201 mV and changed only by 5–10% under the influence of temperature and humidity, while under the same conditions, the short-circuit current was increased 2–3 times. It was concluded that these electrochemical sensors (Al/Gr-Jelly/Cu) can be used as prototypes for the development of jelly electronics.

In a continuation of our efforts to investigate the electrical properties of organic, inorganic, or composite material-based devices [23,24,25], in this paper, we present flexible tactile sensors. These sensors are based on carbon nanotube–glue composite gel and investigated for impedimetric and capacitive tactile pressure and displacement sensing with the plane and semi-cylindrical flexible conductive plates. The fabrication of the sensors is simple and cost-effective. The materials used for the fabrication of these sensors are environmentally friendly. The fabricated sensors may be used for various industrial applications and for demonstration purposes in education.

## 2. Results and Discussions

**Figure 1:**
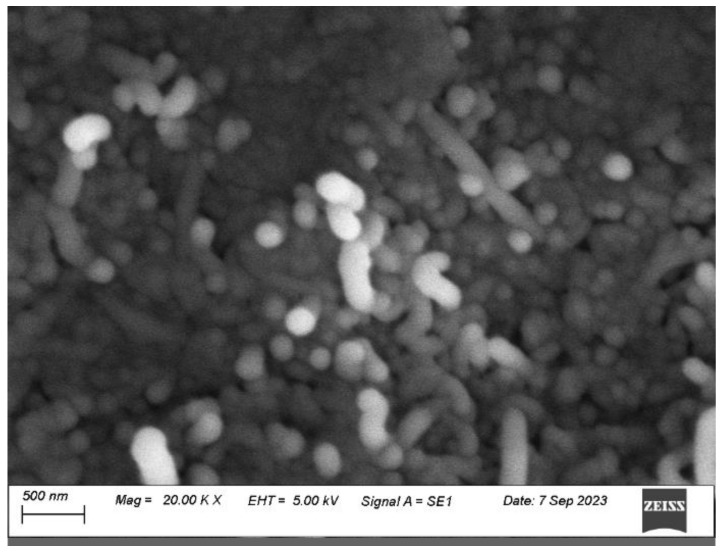
Scanning electron micrograph of the carbon nanotube–glue gel-based electrode.

The scanning electron micrograph of the carbon nanotube–glue gel electrodes is shown in Figure 1. These electrodes were deposited on the rubber substrates using a rubbing-in technique. The CNTs were thoroughly distributed and randomly oriented. In addition to forming a layer on the surface of the rubber substrate, the CNTs also penetrated the substrate, which is depicted by their vertical orientation. This structure makes the CNT–glue electrode stable and reliable.

**Figure 2:**
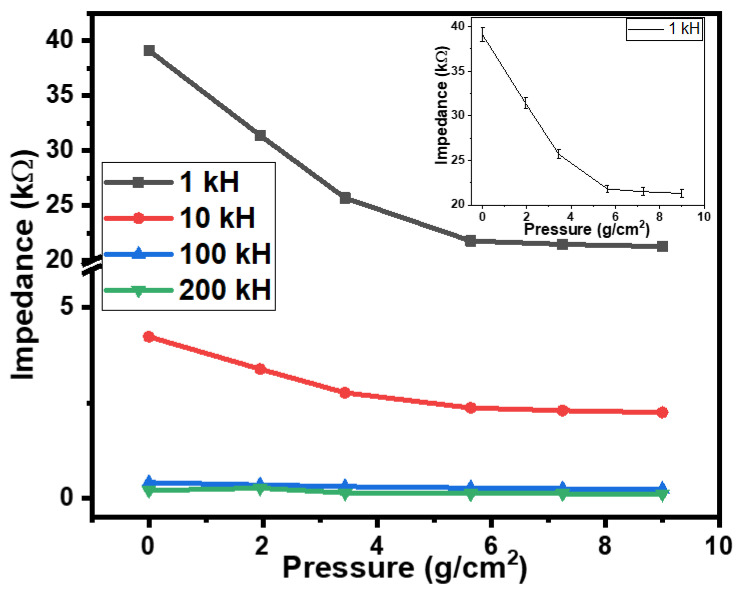
The dependences of the impedances of the flexible rubber–CNT–glue gel-based tactile sensor on the pressure at various frequencies; the inset shows the impedance–pressure relationship with error bars.

**Figure 3:**
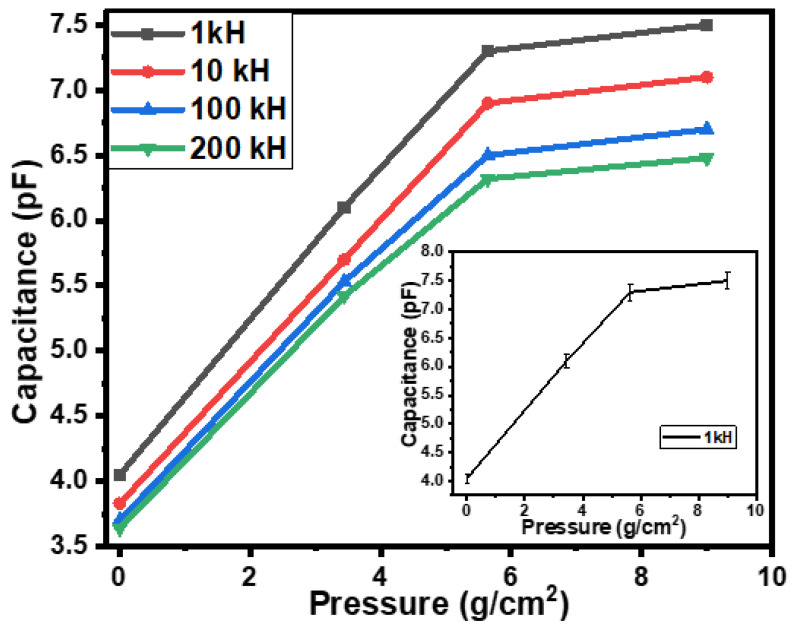
The dependences of the capacitances of the flexible rubber–CNT–glue gel-based tactile sensor on the pressure at various frequencies, and the inset shows the capacitance–pressure relationship with error bars.

Pressure measurements are of prime importance in numerous fields, including health monitoring, process control, environmental monitoring, etc. Fabricated flexible rubber–CNT–glue gel-based sensors were also characterized for their tactile pressure sensing. The sensors were tested under the effect of pressure ranging from 0 to 9.0 g/cm^2^. Figure 2 shows the dependence of the impedances of the tactile sensor on the applied pressure. On applying downward pressure on the semi-cylindrical metallic body of the tactile sensor, the impedance of the sensor decreased by 1.84, 1.88, 1.75, and 1.71 times, respectively, at the frequencies of 1 kHz, 10 kHz, 100 kHz, and 200 kHz. This decrease in impedance could be credited to the increase in the nearness of the flexible metallic body to the CNT–glue gel conductive layers of the tactile sensor. Moreover, the initial values of the impedance were 39.1 MΩ, 4.24 MΩ, 0.415 MΩ, and 0.204 MΩ, correspondingly, at frequencies of 1 kHz, 10 kHz, 100 kHz, and 200 kHz. The experiments were repeated 3 to 4 times. The experimental errors were calculated using the obtained data. For impedance–pressure relationships, the value of the experiment was ±2.0% on average. The inset of Figure 3 shows the impedance–pressure relationship with error bars.

Figure 3 shows the dependence of the capacitance of the tactile sensor on the applied pressure. On increasing the pressure on the semi-cylindrical metallic body, the capacitance increased by 1.86, 1.85, 1.81, and 1.78 times, accordingly, at 1 kHz, 10 kHz, 100 kHz, and 200 kHz. This increase in capacitance occurred due to the change in the position of the metallic body (which, due to downward pressure, came closer to the conductive CNT–glue gel layers of the sensor). Moreover, was also observed that under the influence of pressure, the initial rate of change in impedance and/or capacitance was higher compared to the rate of change in impedance and/or capacitance at higher levels of pressure. When the pressure increased from 0 to 5.6 g/cm^2^ (low-pressure range), the rate of change in capacitance at the frequency of 1 kHz was 0.6 pF-cm^2^/g, which decreased at a higher-pressure range (5.6 g/cm^2^ to 9.0 g/cm^2^) of up to 0.06 pF-cm^2^/g. The rates of change in impedance at the frequency of 1 kHz were 3.07 kΩ cm^2^/g and 0.12 kΩ cm^2^/g, respectively, in the low and high range of pressure. The experimental errors calculated using the obtained data of capacitance–pressure relationships of the fabricated sensors were also up to ±2.0% on average. The inset of Figure 3 shows the capacitance–pressure relationship with error bars.

**Figure 4:**
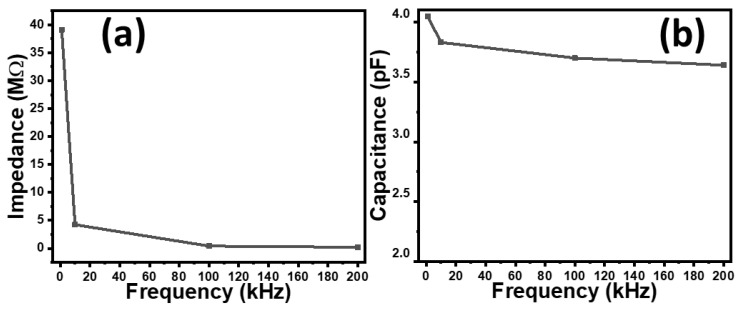
The frequency dependence of the impedance (**a**) and capacitance (**b**) of the fabricated rubber–CNT–glue gel-based tactile sensor.

The results shown in Figure 2 and Figure 3 depict how the response of the sensors also depends on the frequency as well. Therefore, the frequency dependence of the impedance of the fabricated sensors was investigated, as shown in Figure 4a. It is observed that as the frequency increases from 1 kHz to 200 kHz, the impedance sharply falls to approximately 200 times. This behavior of the sensor can be used as a low pass or high pass filter depending on the connection of the sensor, either parallel or in series in the circuit.

The effect of frequency on the capacitance of the sensors was also investigated. Figure 4b shows the dependence of the capacitance of the CNT–glue gel-based sensors on the frequency. It can be seen that, practically, the capacitance does not depend on the frequency.

**Figure 5:**
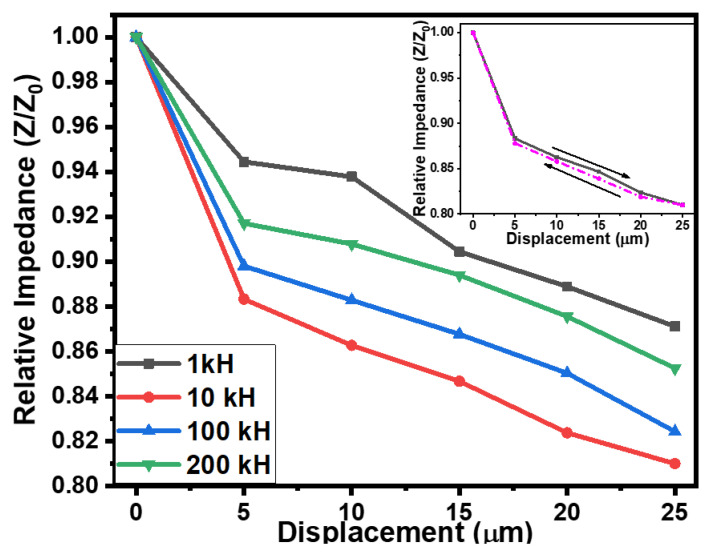
The dependences of the impedances of the flexible rubber–CNT–glue gel = based tactile sensor on the displacement at various frequencies and inset, showing the hysteresis of the impedance–displacement relationship.

**Figure 6:**
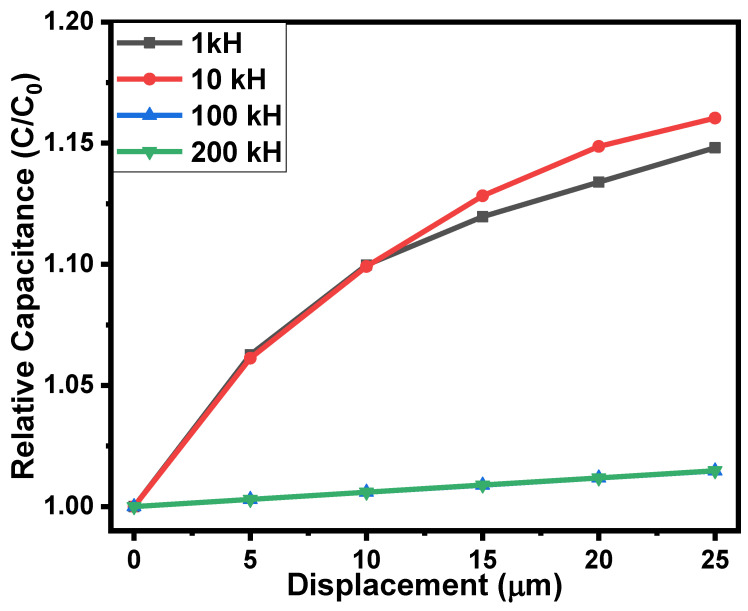
The dependences of the capacitance of the flexible rubber–CNT–glue gel-based tactile sensor on the displacement at various frequencies.

The micro-scale movement or displacement of a surface/object in a specific direction (while in contact with the sensor) can be determined using a tactile displacement sensor. Fabricated rubber–CNT–glue, gel-based sensors were also tested for tactile displacement sensing using a flexible semi-cylindrical conductive body. The displacement of the semi-cylindrical body was controlled via the application or relief of the force on it. The fabricated sensors were tested in the displacement range of 0 to 25 µm. Figure 5 shows the effect of displacement on the impedance and the capacitance of the rubber–CNT–glue gel-based sensors at frequencies ranging from 1 kHz to 200 kHz. On increasing the displacement from 0 to 25 µm, the decrease in impedance of the sensors was noted up to 19%. The impedimetric sensitivity of the fabricated tactile sensor was up to 232 × 10^3^ Ω/µm. On the other hand, the capacitance of the sensors was increased upon increasing the displacement of the semi-cylindrical body towards the sensor. As shown in Figure 6, on increasing the displacement from 0 to 25 µm, the capacitance of the sensor increased up to 15%. This increase in the capacitance of the sensors may be credited to the closeness of the conductive plate with the conductive layers of the CNT–glue composite gel. The results of the impedimetric and capacitive sensitivities of the flexible rubber–CNT–glue, gel-based tactile sensor under the effect of pressure and displacement at various frequencies are presented in Table 1. The displacement-sensing experiments were repeated 3 to 4 times. The experimental errors were calculated using the obtained data. For both impedance–displacement and capacitance–displacement relationships, the value of experimental errors was found to be ±1.5% to 2.0% on average. Moreover, the sensors showed a very small hysteresis in tactile displacement sensing. As shown in the inset of Figure 5, hysteresis is in the range of experimental error.

**Figure 7:**
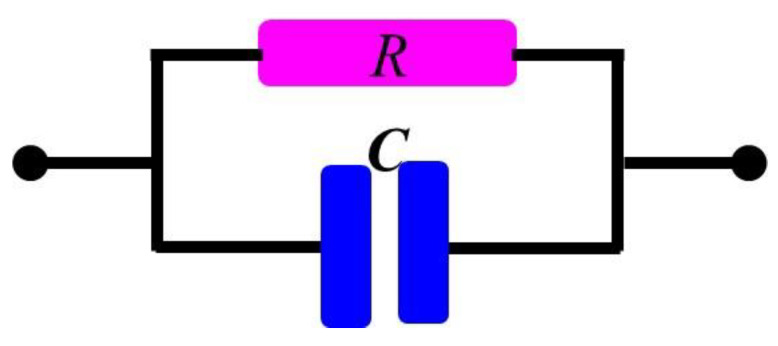
Equivalent circuit of the flexible rubber–CNT–glue gel-based tactile sensor as parallel-connected resistance and capacitance.

Figure 7 shows the equivalent circuit of the sensors as parallel-connected resistance and capacitance. The physically obtained results of the sensors can be explained via the reduction in resistances and enhancement of the capacitance due to the compression of CNT particles under the effect of pressure or displacement. The results shown in Figure 2, Figure 3, Figure 5 and Figure 6 may be explained by the impact of geometrical parameters, mainly the reduction in the gap between the CNT particles of CNT–glue gel layers and an increase in their contact area. Physical parameters such as the charge concentration and the mobility of the charges may also be enhanced in the CNT–glue gel layer under the effect of displacement or pressure. Moreover, the presence of the semi-cylindrical metallic body affects the capacitance and the impedance of the sensor by reducing the gap between the CNT–glue gel layers of the sensors.

As sensing devices, tactile sensors establish the foundation of rapidly growing intelligent systems [26]. In the prospect of intelligent systems, tactile sensors have outstanding applications in various fields, for instance, in medical treatment, wearable devices, robotics, artificial limbs, etc. To display the sensed displacement, a method was patented by Matsunaga et al. [27]. In this method, a single-dimensional imaging device was used to collect the data of single-dimension light distribution. An angular displacement electrical sensor was patented by Nicol as well [28]. This sensor contained one strain gauge (at least) secured to a flexible strip that could bend on a small radius without permanent plastic deformation. Moreover, the sensor’s output was proportional (directly) to the relative angular orientation of the strip’s ends. The optical displacement of the sensor’s properties was also described by Jakson et al. [29]. Xue et al. [30] patented a long-range displacement passive wireless sensor that consisted of a substrate, a sloping channel ground plate, and a chipped circular patch antenna with two opened windows of rectangular shape. The lack of bonding between the ground plate and antenna allowed the antenna (chipped) to slide along the slopping channel. An overview of the presented information published in a number of papers shows that the CNT–glue gel-based impedimetric and capacitive tactile pressure and displacement flexible sensor’s structure, materials, and properties were presented for the first time in this study.

The simulation of the obtained experimental was conducted using mathematical functions. The experimental results presented in Figure 2, Figure 3, Figure 5 and Figure 6 may be simulated using the following mathematical function [31]:

(1) $$f\left(x\right)=e^{x}$$

**Figure 8:**
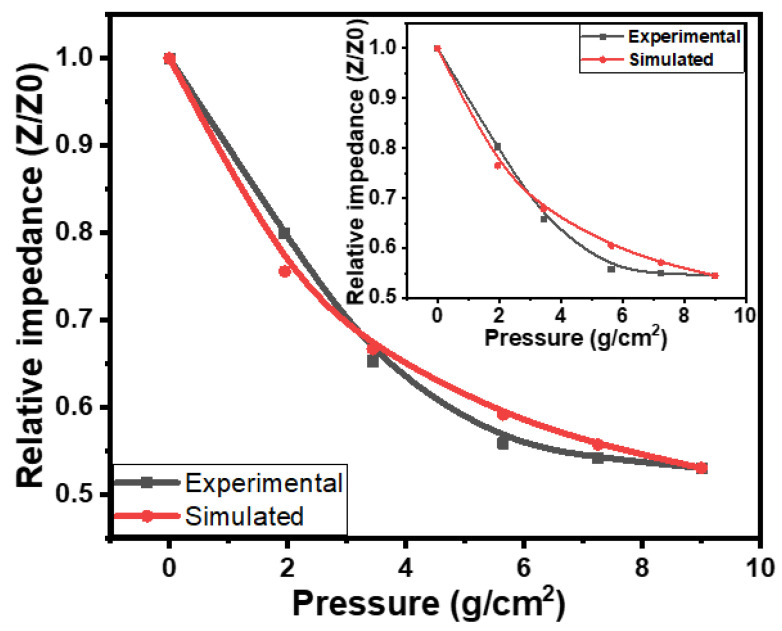
Comparison of the simulated and experimental impedance–pressure relationships of flexible rubber–CNT–glue gel-based tactile sensors at 10 kHz and 1 kHz (inset).

For the simulation of different electrical parameters, the above function (Equation (1)) can be modified. For the impedance–pressure relationships of flexible rubber–CNT–glue gel-based tactile sensors, the above mathematical function can be modified as follows:

(2) ZZ0=e∆P∗k1(∆Pm0.65∆P+0.35∆Pm)

where *Z* and *Z*_0_ are the instantaneous and initial impedances of the flexible rubber–CNT–glue gel=based tactile sensors, respectively. The Δ*P_m_* and Δ*P* are the maximum and instantaneous change in pressure, consequently. *k*_1_ is the impedance pressure constant, and its value is −7.04 × 10^−2^ cm^2^/g. The simulated and experimental impedance–pressure relationships of flexible rubber–CNT–glue gel-based tactile pressure sensors are compared in Figure 8. Figure 8 shows the comparison of the experimental and simulated results of the sensors at a frequency of 10 kHz, while the inset in Figure 8 shows the comparison of these results at the frequency of 1 kHz. The simulated results illustrate good matching with the experimental results. The consistency of the simulated results with experimental results is with an accuracy of 97 ± 0.25% on average.

**Figure 9:**
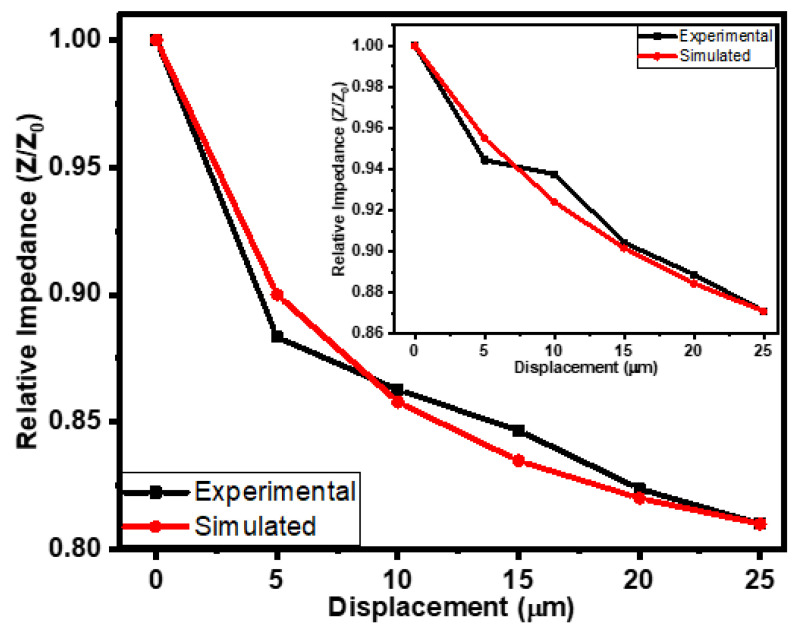
Comparison of the simulated and experimental impedance–displacement relationships of flexible rubber–CNT–glue gel-based tactile sensors at 10 kHz and 1 kHz (inset).

For the simulation of the impedance–displacement relationships of the flexible rubber-CNT-glue gel-based tactile sensors at various frequencies, Equation (1) was modified as follows:

(3) ZZ0=e∆d∗k2(∆dm0.75∆d+0.25∆dm)

where Δ*d* and Δ*d_m_* are the instantaneous and the maximum change in the compressive displacement of the metallic body towards the flexible rubber–CNT–glue gel-based tactile sensor, respectively. The initial and the instantaneous impedances of flexible rubber–CNT–glue gel-based tactile sensors are denoted by Z_0_ and Z, accordingly. *k_2_* represents the impedance displacement constant with value of −8.43 × 10^−3^ µm^−1^. Figure 9 shows the comparison of the experimental and simulated impedance–displacement relationships of the flexible rubber–CNT–glue gel-based tactile sensors at the frequencies of 10 kHz and 1 kHz (inset). The simulated results of impedance–displacement relationships are in good agreement with the experimental results. They illustrated a good match with the experimental results. The consistency of simulated results with experimental results was 98 ± 0.25% on average.

## 3. Conclusions

The results of fabricated carbon nanotube–glue composite gel-based surface-type elastic deformable capacitive tactile pressure and compressive displacement sensors with a cylindrical shape deformable (flexible) metallic body are presented. The obtained results were explained on the base of changes occurring in the shape and geometrical parameters of the cylindrical-shaped conductive body and the distance between the conductive plates of the capacitive sensors during the compressing processes which took place under the effect of applied pressure or compressive displacement. It was shown that the design and the fabrication of the sensors are simple and cost-effective, while its utilization is also earthy. Moreover, the materials used for the fabrication of these sensors are environmentally friendly. Fabricated sensors may be used for various industrial applications and for demonstration purposes in education.

## 4. Experimental Section

**Figure 10:**
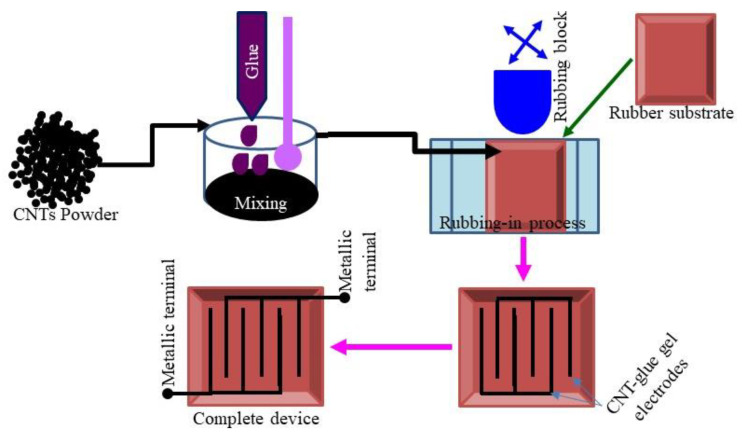
Simplified schematic diagram showing the fabrication steps of deformable rubber–CNT–glue gel-based impedimetric and capacitive tactile sensors for the measurement of pressure and compressive displacement.

For the fabrication of carbon nanotube–glue composite gel-based surface-type tactile pressure sensors, a multiwalled carbon nanotube (CNT) powder was purchased from Sun Nanotek Co., Ltd. (Nanchang, China) (available online: http://www.sunnano.com/cnt%20product.html, accessed on 12 August 2022). The diameter and the length of the CNTs were in the range of 30 to 100 nm and 2 to 3 µm, respectively. The glue used was produced by UHU, GmbH & Co. KG Hermannstraße 7D—77815—Buhl/Baden, Karlsruhe, Germany (available online: www.UHU.com, accessed on 17 December 2021). The commercially available plastic sheets were used as a substrate. The surface sizes of the substrates were equal to 5 cm × 5 cm. The gel of the CNT and glue composite was prepared by mixing 60 wt.% CNT and 40 wt.% glue using a mortar and pestle. The prepared composite gel was used to deposit conductive layers (CNT–glue gel) electrodes. The deposition of the conductive layers was performed using the rubbing-in technique. The thickness of the deposited layers was in the range of 21 to 25 µm, while the gap between the two gel layer electrodes was equal to 3 mm on average. The total surface sizes of the rubber–CNT–glue gel-based impedimetric and capacitive tactile sensors were equal to 5 cm and 5 cm. The simplified schematic diagram of fabrication steps and top view of the fabricated rubber–CNT–glue gel-based elastic impedimetric and capacitive tactile pressure and displacement sensors is shown in Figure 10.

**Figure 11:**
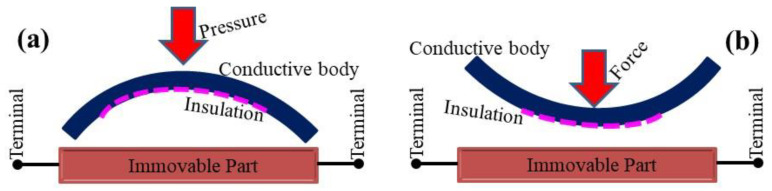
Schematic drawing (front view) of the fabricated deformable rubber–CNT–glue gel-based impedimetric and capacitive tactile sensors in pressure (**a**) and displacement (**b**) sensing modes: the immovable part comprises a plastic substrate covered by CNT and glue composite (60 wt.% to 40 wt.%) conductive line electrodes (as shown in Figure 10); the conductive body consists of a flexible conductive semi-cylindrical plate which contains insulation (thin layered) at various points to avoid direct electrical contact with CNT–glue gel electrodes; metallic terminals are indicated for the connection to measure the changes in the electrical parameters while the arrows show the direction of applied pressure or force on the sensor.

Figure 11 shows the schematic diagram (front view) of the fabricated deformable rubber, CNT–glue-based tactile pressure, and compressive displacement sensor, along with the direction of the applied pressure or force. The semi-cylindrical metallic plate is shown in Figure 11 and plays the role of a flexible conductive body in the tactile sensor. It contains insulation (thin-layered) at various points to avoid direct electrical contact with the CNT–glue gel electrodes. The movement of this metallic body causes the variation in impedance and capacitance in the rubber–CNT–glue gel-based sensor. These changes in capacitance are measured by connecting the LCR meters with the metallic terminals.

For the morphological analysis of rubber–CNT–glue gel, the EVO-15 Scanning Electron Microscope (Carl Zeiss, Oberkochen, Germany) was used. For the electrical measurements, a digital multi-meter MT 4090 LCR (MOTECH) was used. The changes in the impedance and the capacitance of the sensors were measured in the frequency range of 1 kHz to 200 kHz. All the testing was conducted at room temperature and in an ambient environment. The fabricated sensors were tested by applying pressure on the semi-cylindrical plate. The terminals of the sensors (shown in Figure 11) were connected to the LCR meter MT 4090 LCR. A detailed construction of the experimental setup will be provided elsewhere.

## Figures and Tables

**Table 1:** The results of the impedimetric and capacitive sensitivities of the flexible rubber–CNT–glue gel = based tactile sensor under the effect of pressure and displacement at various frequencies.

Device	Type of Sensitivity	Frequency (kHz)			
		1	10	100	200
Pressure sensor	Impedimetric (Ω cm^2^/g)	197.7 × 10^4^	221.1 × 10^3^	198.0 × 10^2^	94.4 × 10^2^
	Capacitive (pF cm^2^/g)	3.83 × 10^−1^	3.63 × 10^−1^	3.33 × 10^−1^	3.15 × 10^−1^
Displacement sensor	Impedimetric (Ω/µm)	232.0 × 10^3^	332.0 × 10^2^	324.0 × 10^2^	128.0 × 10^1^
	Capacitive (pF/µm)	2.1 × 10^−2^	2.2 × 10^−2^	2.0 × 10^−3^	1.99 × 10^−2^

## Data Availability

The data presented in this study are openly available in article.
